# Overview and quality assurance for the oral health component of the National Health and Nutrition Examination Survey (NHANES), 2011–2014

**DOI:** 10.1186/s12903-019-0777-6

**Published:** 2019-05-29

**Authors:** Bruce A. Dye, Joseph Afful, Gina Thornton-Evans, Timothy Iafolla

**Affiliations:** 1 National Institutes of Health, National Institute of Dental and Craniofacial Research, 31 Center Drive Suite 5B55, Bethesda, MD USA; 2 Peraton Corporation, as a contractor for the Centers for Disease Control and Prevention, National Center for Health Statistics, Hyattsville, MD USA; 3 Centers for Disease Control and Prevention, National Center for Chronic Disease Prevention and Health Promotion / Division of Oral Health, Atlanta, GA USA

**Keywords:** NHANES, Oral health, Data reliability, Epidemiology, Surveillance, Quality assurance, Dental public health

## Abstract

**Background:**

Following implementation in 2009–2010 to the oral health component for the National Health and Nutrition Examination Survey (NHANES), a full-mouth periodontal examination was continued during 2011–2014. Additionally, a comprehensive dental caries assessment was re-introduced in 2011 after a 6-year absence from NHANES. This report provides oral health content information and results of dental examiner reliability statistics for key intraoral assessments conducted by dentists during 2011–2014.

**Methods:**

During the 2011–2014 NHANES 17,463 persons age 1 and older representing the US civilian, non-institutionalized population received an oral health examination. From this group, 387 individuals underwent a repeat examination conducted by the survey reference examiner. A combination of examiner training and calibration, electronic data capture, and ongoing performance evaluation with statistical monitoring was used to ensure conformance with NHANES protocols and data comparability to prior data collection periods.

**Results:**

During 2011–2014, the Kappa statistics for the tooth count assessment ranged from 0.96 to 1.00, for untreated dental caries Kappa scores were 0.93 to 1.00. The overall Kappa statistics for identifying combined moderate-severe periodontitis using the CDC/AAP case definition was 0.66 and 0.69 with percent agreement of 83 to 85% during 2011–2014. When evaluating inter-examiner agreement using information collected from 3 periodontal sites for comparability to the NHANES 2003–04 periodontal examination protocols, Kappa scores for combined moderate-severe periodontitis was 0.65 and 0.80 during 2011–2014. For total mean attachment loss and pocket depth across all 6 periodontal sites, the inter-class coefficients (ICCs) ranged from 0.80–0.90 and 0.79–0.86 respectively. Site-specific mean attachment loss ICCs were generally higher for the 4 interproximal measurements compared to the 2 mid-site probing measurements and this observation was similar in 2009–2010.

**Conclusion:**

During 2011–2014, results overall indicate a high level of data quality and substantial examiner reliability for tooth count and dentition; reliability for periodontal disease, across various assessments, was at least moderate. When comparing the 2011–2014 examiner performance to findings from 2003 to 2004, comparable concordance between the examiners and the reference examiner exists.

## Background

For the past fifty years, a number of health examination surveys of the US population have been conducted by Centers for Disease Control and Prevention (CDC), National Center for Health Statistics (NCHS). Between 1959 and 1994, these health examinations were periodic. The earlier surveys were known as the National Health Examination Survey (NHES) and were later renamed the National Health and Nutrition Examination Survey (NHANES) when a dietary assessment was added in 1970. Since 1999, NHANES has been a continuous, annual survey. The current NHANES is a nationally representative sample for each year of data collection but data are released in two-year periods to protect confidentiality and increase statistical reliability. Some of the primary objectives of NHANES include: monitoring the prevalence, awareness, treatment and control of selected diseases and health conditions in the U.S. and assessing factors that affect morbidity, mortality and quality of life.

NHANES is the main source of information on oral health (OH) status in the US. This includes data on tooth loss, dental caries, and periodontal disease among other OH conditions. Since 1999, a few novel OH assessments have been introduced to NHANES. Information has been collected on dental erosion and tooth wear, functional occlusal contacts, and OH related quality of life [1]. Using surplus sera, antibodies to nearly two dozen bacteria, including established periodontal pathogens, have been quantified using checkerboard immunoblotting [2] and periodontal status was evaluated with a full-mouth probing of six periodontal sites around each tooth [3, 4]. More recently, information on home water fluoride and plasma levels for youths were collected and a unique imaging approach was used to assess for dental fluorosis [5, 6].

During the past decade, NHANES has made some important contributions to the methodology of OH surveillance. A basic screening exam (BSE) was conducted between 2005 and 2008 and was modeled on a dental caries and sealant screening protocol developed by the American Association of State and Territorial Dental Directors [7, 8]. The BSE was designed to be used in epidemiological settings to collect subject-level information when resources are limited and dentists are not available to conduct the screenings. Another important methodological contribution to OH surveillance that has occurred with NHANES has been the exploration of alternative approaches to the typical resource-intensive examination for periodontal diseases. This has contributed to the development of standard case definitions for surveillance of periodontitis [9, 10] and the development and evaluation of self-report measures for predicting prevalence of periodontitis [11, 12]. These efforts also have contributed to international recognition of the need for basic reporting guidelines for periodontal disease measures in epidemiologic studies [13].

Quality assurance processes for national surveys are critically important for the collection of accurate data given that the information originating from these data is frequently used to inform national health policy discussion and legislation. The implementation and reporting of systematic quality assurance procedures is needed to minimize errors in data collection and improve data reliability and transparency, which facilitate the generation of valid findings and the acceptability of results by the public [14]. NHANES is an important source for nationally representative oral health data. The simultaneous collection of hundreds of other health data points, including physical measurements, diet, laboratory findings, and genetic samples, enables researchers to develop and test hypotheses on a plethora of health-related issues. Equally important, these data are very useful for assessing changes in population health status over time. The aim of this methodological paper is to describe important aspects of the 2011–2014 NHANES oral health examination and to report on the quality assurance (QA) program by examining data quality, including statistical analyses of reliability for the dentition and periodontal assessments.

## Methods

### NHANES 2011–2014 overview

The civilian, non-instutionalized population living in the 50 United States and the District of Columbia was the target population for NHANES 2011–2014. The survey used a stratified, multistage probability sampling design to select participants. The 2011–2014 sampling domains were defined by age group, sex, low-income status, and race and Hispanic origin. As part of the sample design, the survey over samples some population subgroups to facilitate the calculation of more precise estimates for these groups. Over sampling for the 2011–2014 survey included all Hispanics, non-Hispanic Blacks, non-Hispanic Asians, some low-income persons, and non-Hispanic white and other persons 80 years and older. Sample design characteristics for NHANES 2011–2014 are presented in Table 1. Informed consent was obtained for all participants and all data collection protocols are approved by the NCHS Research Ethics Review Board. Additional information including protocol numbers are available here: https://www.cdc.gov/nchs/nhanes/irba98.htm

**Table 1:** Sampling Design Characteristics for National Health and Nutrition Examination Survey (NHANES), 2011–14

Characteristic	NHANES 2011–12	NHANES 2013–14
Age of the target population	From birth	From birth
Dental Exam Age	1 year and older	1 year and older
Number of survey locations	30	30
Eligible geographical area for sample	50 states + DC	50 states + DC
Groups target for oversampling	Persons 60 years and older; all Hispanics; non-Hispanic Blacks; non-Hispanic Asians; and low-income whites	Persons 60 years and older; all Hispanics; non-Hispanic Blacks; non-Hispanic Asians; and low-income whites
All ages interviewed in the Home	9756	10,175
All ages examined in the MEC	9338	9813
All persons 1 year and older MEC examined	8956	9422
All persons 1 year and older Oral Health examined	8472	8991

Notes

[DC]: District of Columbia

[MEC]: Mobile Examination Center

As in previous NHANES, participants are interviewed in their homes and then given a complete health examination, which includes the collection of biologic specimens for lab testing at a mobile examination center (MEC). Following household identification and the administration of a screening questionnaire by field interviewers to determine participant eligibility, informed consent is obtained for the home interview. Trained interviewers conduct the home interview, which has two main components: the household and sample person interviews. Following completion of the home interview, interviewed participants are asked to participate in a health examination in a MEC and undergo a second series of informed consent procedures for the health examination. Additional information on the background and content of NHANES can be found at: http://www.cdc.gov/nchs/about/major/nhanes/datalink.htm.

### NHANES 2011–2014 Oral health component

The 2011–2014 OH component was comprised of information collected during the home interview, the MEC examination, and specimens for lab analyses. The home interview OH questions covered topics such as dental visit frequency, perceived OH status, the receipt of preventive health information, oral pain, periodontal disease and oral cancer. For 2013–2014, a sample of tap water from the home of persons age 19 and younger was collected to test for fluoride level. Although sampled persons age 1 and older were generally eligible for the OH interview and exam in 2011–2014, specific OH assessments were age-based and eligibility varied among study participants (Table 2).

**Table 2:** Age eligibility for Oral Health assessments, NHANES 2011–2014

Assessment	2011–2012	2013–2014
	age in years	age in years
Home Interview		
Dental health perception, dental visits and dental care utilization*	1+	1+
Tooth brushing and tooth paste use	–	3–19
Fluoride supplement use	–	3–15
Receipt of health information from a dental professional	16+	16+
Oral pain and quality of life*	30+	30+
Oral cancer	30+	30+
Periodontal Disease	30+	30+
MEC Exam		
Medical history screening	30+	30+
Tooth count	1+	1+
Dental caries	1+	1+
Dental sealants	3–19	3–19
Dental fluorosis	6–19	6–19
Periodontal assessment	30+	30+
Recommendation for care	1+	1+
Laboratory		
Fluoride in plasma	–	3–19
Fluoride in water	–	birth-19

For 2011–2014, the MEC oral health exam was performed by dentists with a dental license in at least one U.S. jurisdiction and trained in NHANES survey methods. To assist with data entry, other MEC personnel were trained as dental recorders. NHANES has two full-time MEC teams collecting data concurrently with a primary dentist assigned to each team. During 2011–2014, there were three primary dental examiners assigned to the MEC teams and they performed nearly 94% of all dental examinations in 2011–2014. All dental examiners performing OH examinations in this four-year period were trained and calibrated by Dr. Dye (author), as were the examiners in the 1999–2004 NHANES cycle. All dental examinations took place in a designated room inside the MEC that includes a portable dental chair, light, and compressed air, and digital imaging equipment.

If the sampled person was age 30 or older, a series of health screening questions were administered by the dentist to determine eligibility for the periodontal examination. If a participant acknowledged having a heart transplant, an artificial heart valve, congenital heart disease, or ever having bacterial endocarditis, they were excluded from periodontal probing. In 2011–2014, approximately 4% of the assessed adults were excluded from the periodontal examination because of medical history concerns.

For all sample persons age 1 and older, the first OH assessment began with “tooth count” to identify the presence or absence of permanent and/or primary teeth, including retained dental root tips and dental implants. The second assessment was to identify which teeth had been affected by the dental caries process, which could later be used to help create the classic “DMFT” score (decayed, missing, and filled teeth). Following the caries assessment, evaluations for the presence of dental sealants and dental fluorosis were completed for children and adolescents. Additionally, persons age 6–19 undergoing a standard blood draw had a portion of their blood tested for plasma fluoride concentration in 2013–2014.

Adults aged 30 years and older were eligible for a full-mouth periodontal examination (FMPE) if they had a least one natural permanent tooth present. Gingival recession and pocket depth measures were made at six sites per tooth (the distal-facial (DF), mid-facial (BF), mesio-facial (MF), distal-lingual (DL), mid-lingual (BL), and mesio-lingual (ML)) sites using a HU-Friedy periodontal probe color-coded and graduated in 2-mm increments. All four quadrants were examined, and all measurements were rounded to the lowest whole millimeter. An algorithm in the data entry program calculated loss of attachment (difference between pocket depth and gingival recession). The FMPE protocol was introduced on NHANES in 2009–2010 and was carried over into 2011–2014. Information describing NHANES FMPE protocols and the 2009–2010 data quality is available elsewhere [4]. Detail information regarding data collection protocols for the 2011–2012 and 2013–2014 oral health component is available elsewhere: [https://wwwn.cdc.gov/nchs/data/nhanes/2011-2012/manuals/Oral_Health_Examiners_Manual.pdf and https://wwwn.cdc.gov/nchs/data/nhanes/2013-2014/manuals/Oral_Health_Examiners.pdf].

### Quality assurance

Dental examiners underwent a comprehensive training and calibration period, including periodic monitoring and recalibration, to ensure quality OH data. During the training phase, trainees participated in a comprehensive presentation designed to familiarize them with the study protocols. This slide presentation covered assessment criteria, data recording, infection control procedures, and emergency preparedness guidelines. Following a demonstration examination, a series of standardization sessions were conducted where the reference examiner and trainee examined the same set of approximately 60–65 volunteers. Trainees were encouraged to ask questions during their examinations with feedback provided during each examination round to minimize differences in the application of criteria and coding. Following the standardization sessions, a set of preliminary calibration sessions was performed on an additional 30–35 volunteers and the trainees conducted independent replicate examinations without discussion. Data from these calibration sessions were analyzed to assess consistency between each trainee and the reference examiner. Training typically lasted for 40 h and was conducted in the Washington, DC, metropolitan area. A final calibration session was conducted at the MEC and was performed during normal field operations over a three to four-day period for each examiner.

There are three main activities employed to facilitate the ongoing collection of quality OH data on NHANES. To reduce data recording errors, all data are directly entered into an electronic data management system and automated data management utilities are used to check for out of range values at the time of entry. Project managers periodically observe staff performing data collection to ensure that protocols are followed. Finally, the reference examiner visits each dental examiner approximately twice a year to replicate approximately 25-to-30 random OH examinations during each MEC visit. Data from these replicate exams were used to produce inter-rater reliability statistics to objectively evaluate examiner agreement. Although dental examiners were aware of the inter-rater evaluations being conducted, examiners are blinded to each other’s observations. Because all inter-rater reliability statistics were calculated after the MEC evaluation visits, the findings were unavailable for real time use. However, all examiners did receive general feedback pertaining to their performance immediately following each evaluation visit by the reference examiner.

To ascertain examiner agreement compared to the reference examiner, percent agreement, Kappa statistics, and inter-class correlation coefficients (ICCs) were calculated using SAS software (version 9.3, SAS Institute Inc., Cary, NC). For purposes of this report, reliability statistics were analyzed and presented for tooth count, dental caries and sealants, and periodontal status. The calculation of inter-examiner reliability statistics followed similar procedures used for previous NHANES oral health reliability reports to facilitate comparisons [1, 4, 8, 15]. Kappa statistics calculated for tooth count assessed four indicators: complete tooth loss, retention of all third molars, having at least one retained root tip, and tooth retention, which was the total number of primary and permanent teeth present. Kappa statistics were calculated for detecting at least one tooth with dental caries experience or only untreated caries in either primary dentition or in permanent dentition. For the evaluation of periodontal status, a number of different definitions and categories were used including the CDC Periodontitis Workgroup (CDC/AAP) case definitions, which have been recommended for periodontitis surveillance [9, 10]. In addition to calculating Kappa statistics for categorical periodontal status variables, inter-rater reliability for continuous variables was assessed by comparing ICCs generated from subject-level means (mm) for loss of attachment (AL) and pocket depth (PD) using measurements obtained from all six periodontal sites. Reliability statistics were calculated at the person (or subject) level and are unweighted. All results presented in this report do not incorporate the NHANES sample weights.

## Results

Table 3 shows the number of individuals participating in the home interview, MEC examination, and the OH examination by various characteristics in NHANES 2011–2012 and 2013–2014. Among all participants age 1 and older in 2011–2012, 8956 individuals had a MEC examination and 8472 individuals had an oral examination. More people in 2013–2014 were examined in a MEC (9422) and completed an OH examination (8991).

**Table 3:** Number of Sampled Persons Aged 1 year and older with Interview, MEC and Oral Health Exams by Selected Characteristics, National Health and Nutrition Examination Survey (NHANES) 2011–14

Characteristic	NHANES 2011–2012			NHANES 2013–2014		
	HIQ	MEC	OHX	HIQ	MEC	OHX
Age						
1–5 years	1203	1135	1119	1198	1131	1111
6–11 years	1328	1272	1236	1371	1312	1287
12–19 years	1273	1230	1186	1432	1391	1367
20–34 years	1490	1424	1322	1482	1420	1363
35–50 years	1366	1329	1245	1507	1467	1349
50–64 years	1454	1400	1276	1474	1436	1337
65–74 years	669	637	584	748	733	689
75+ years	581	529	504	558	532	488
Sex						
Male	4663	4463	4247	4802	4637	4458
Female	4701	4493	4225	4968	4785	4533
Race and ethnicity						
Hispanic	2296	2195	2066	2560	2487	2358
Non-Hispanic Black	2588	2492	2358	2186	2121	2029
Non-Hispanic Asian	1257	1191	1104	1046	992	910
Non-Hispanic White	2860	2729	2604	3529	3399	3278
Other Race	363	349	340	449	423	416
Poverty status						
Less than 100% FPG	2593	2480	2331	2581	2487	2380
100–199% FPG	2246	2163	2059	2322	2252	2140
200% or Higher FPG	3720	3571	3402	4119	3985	3822
Education						
Less than high school	1200	1136	1031	1073	1042	944
High school	977	929	868	1086	1047	965
More than high school	2384	2296	2149	2649	2576	2424
Smoking history						
Current Smoker	876	843	780	959	923	856
Former Smoker	1185	1136	1057	1243	1206	1140
Never Smoker	2498	2381	2210	2609	2538	2339
Dental visit past year						
Yes	5713	5474	5205	6142	5939	5717
No	3627	3462	3247	3616	3471	3262
Total	9364	8956	8472	9770	9422	8991

Notes: Education and Smoking are only for sampled persons aged 30 years and older

[HIQ]: Home Interview

[MEC]: Mobile Examination Center

[OHX]: Oral Health Examination

[FPG]: Federal Poverty Guideline

The inter-rater reliability statistics for the two primary dental examiners (A & B) employed during 2011–2012 and 2013–2014 data collection cycles for tooth count, dentition, and periodontal status are shown in Table 4. Examiner A is the same person in both periods (2011–2012 and 2013–2014), whereas examiner B represents a different examiner for each of the two data collection periods. Overall, the Kappa statistics for the tooth count assessment ranged from 0.96 to 1.00 with a percent agreement ranging from 99 to 100% for the combined period during 2011–2014. For any dental caries in the primary dentition, Kappa scores were 0.93 and 1.00 and percent agreement was 97 and 100% during 2011–2014. For any untreated dental caries, the Kappa scores were 0.74 and 1.00 and percent agreement was 90 and 100% during the same period. Between 2011 and 2012 and 2013–2014, Examiner A matched the reference examiner’s results for caries experience in primary teeth. For dental caries in the permanent dentition, Kappa scores were 0.93 and 0.96 and percent agreement was 97 and 98% during 2011–2014. For untreated dental caries, the Kappa scores were 0.82 and 0.91 and percent agreement was 94 and 97% during the same period. The results from Examiner A regarding replicating the reference examiner for caries experience in the permanent dentition was 0.94 and 0.86 between 2011 and 2012 and 2013–2014 respectively. Overall, the Kappa statistics for dental sealant prevalence was 0.85 to 0.88 during 2011–2014. Examiner reliability was lower in 2011–2012 compared to 2013–2014, with Kappa scores of 0.67 and 0.75 in 2011–2012, and improving to 1.00 for both examiners in 2013–2014.

**Table 4:** Inter-rater Reliability Statistics (Kappa) for Selected Oral Health Conditions, National Health and Nutrition Examination Survey (NHANES) 2011–2014

**#**	Assessments	NHANES 2011–2012				NHANES 2013–2014				NHANES 2011–2014			
		n	%	kappa	ASE	n	%	kappa	ASE	n	%	kappa	ASE
	Tooth Count												
A	Edentulism	76	100.00	1.00	0.00	101	100.00	1.00	0.00	177	100.00	1.00	0.00
B	Edentulism	88	100.00	1.00	0.00	122	100.00	1.00	0.00	210	100.00	1.00	0.00
A	Tooth Retention	76	98.68	0.97	0.03	101	100.00	1.00	0.00	177	99.44	0.99	0.01
B	Tooth Retention	88	100.00	1.00	0.00	122	99.18	0.98	0.02	210	99.52	0.99	0.01
A	1 or more retained root tips	76	100.00	1.00	0.00	101	100.00	1.00	0.00	177	100.00	1.00	0.00
B	1 or more retained root tips	88	100.00	1.00	0.00	122	99.18	0.98	0.02	210	99.52	0.97	0.03
A	Have all 4 third molars	76	98.68	0.93	0.07	101	100.00	1.00	0.00	177	99.44	0.96	0.04
B	Have all 4 third molars	88	100.00	1.00	0.00	122	99.18	0.98	0.02	210	99.52	0.96	0.04
	Dentition												
A	Untreated caries primary teeth	15	86.67	0.66	0.22	36	91.67	0.77	0.13	51	90.20	0.74	0.11
B	Untreated caries primary teeth	29	100.00	1.00	0.00	33	100.00	1.00	0.00	62	100.00	1.00	0.00
A	Caries experience primary teeth	15	100.00	1.00	0.00	36	100.00	1.00	0.00	51	100.00	1.00	0.00
B	Caries experience primary teeth	29	100.00	1.00	0.05	33	93.94	0.88	0.08	62	96.77	0.93	0.05
A	Untreated caries permanent teeth	71	98.59	0.96	0.04	84	98.81	0.97	0.03	155	96.77	0.91	0.04
B	Untreated caries permanent teeth	77	98.70	0.93	0.07	114	94.74	0.88	0.05	191	94.24	0.82	0.05
A	Caries experience permanent teeth	71	97.18	0.94	0.04	84	95.24	0.86	0.07	155	98.07	0.96	0.02
B	Caries experience permanent teeth	77	100.00	1.00	0.00	114	91.23	0.77	0.07	191	96.86	0.93	0.03
A	Has dental sealants	31	90.32	0.67	0.18	29	100.00	1.00	0.00	60	95.00	0.85	0.08
B	Has dental sealants	24	87.50	0.75	0.14	28	100.00	1.00	0.00	52	94.23	0.88	0.06
	Periodontal												
A	Moderate + Severe Periodontitis	25	84.00	0.66	0.15	38	81.58	0.61	0.13	63	82.54	0.66	0.09
B	Moderate + Severe Periodontitis	35	77.14	0.55	0.13	51	90.20	0.79	0.09	86	84.88	0.69	0.08
A	Any Periodontitis	25	84.00	0.66	0.15	38	78.95	0.56	0.13	63	82.54	0.66	0.09
B	Any Periodontitis	35	77.14	0.55	0.13	51	88.24	0.75	0.09	86	84.88	0.69	0.08
A	Periodontal Disease ^SR11^	25	76.00	0.48	0.18	38	76.32	0.45	0.15	63	76.19	0.52	0.10
B	Periodontal Disease ^SR11^	35	77.14	0.51	0.14	51	80.39	0.60	0.11	86	79.07	0.58	0.09
A	1 site with > = 4 mm AL	25	92.00	0.82	0.12	38	76.32	0.53	0.14	63	82.54	0.64	0.10
B	1 site with > = 4 mm AL	35	77.14	0.51	0.14	51	76.47	0.52	0.11	86	76.74	0.52	0.09
A	1 site with > = 6 mm AL	25	80.00	0.58	0.17	38	89.47	0.71	0.14	63	85.71	0.66	0.10
B	1 site with > = 6 mm AL	35	85.71	0.66	0.14	51	86.28	0.61	0.13	86	86.05	0.64	0.09
A	1 site with > = 5 mm PD	25	76.00	0.48	0.16	38	92.11	0.69	0.17	63	85.71	0.59	0.12
B	1 site with > = 5 mm PD	35	97.14	0.92	0.08	51	88.24	0.60	0.15	86	91.86	0.74	0.09
A	1 site with > = 7 mm PD	25	100.00	1.00	0.00	38	100.00	1.00	0.00	63	98.41	0.79	0.20
B	1 site with > = 7 mm PD	35	94.29	0.48	0.31	51	96.08	0.98	0.01	86	95.35	0.31	0.25
A	Moderate + Severe Periodontitis ^3 sites^	25	80.00	0.60	0.15	38	86.84	0.65	0.14	63	84.13	0.65	0.10
B	Moderate + Severe Periodontitis ^3 sites^	35	91.43	0.79	0.12	51	92.16	0.80	0.09	86	91.86	0.80	0.07
A	Periodontal Disease ^3 sites^	25	92.00	0.82	0.12	38	89.47	0.61	0.17	63	90.48	0.73	0.10
B	Periodontal Disease ^3 sites^	35	77.14	0.52	0.15	51	84.31	0.61	0.12	86	81.40	0.58	0.09

Notes

[n]: Number of replicate exams

[%]: Percent agreement

[ASE]: Asymptotic standard error (kappa)

[#]: Dental examiner identification

[AL]: Attachment loss

[PD]: Pocket depth

[SR11]: Using earlier CDC/NCHS Series 11 Report definition

[3 sites]: Based on partial-mouth design used in earlier NHANES

During 2011–2014, the overall Kappa statistics for identifying combined moderate-severe periodontitis using the CDC/AAP case definition was 0.66 to 0.69 for examiners A and B with percent agreement of 83 to 85%. When assessing for any periodontitis, the inter-examiner results ranged from 0.55 to 0.75 during 2011–2012 and 2013–2014. When applying the case definition for periodontal disease used in the published Series 11 Report [13], inter-examiner reliability was similar for both examiners (Kappa 0.52 and 0.58, percent agreement 76 and 79%). The Kappa scores for 4 mm and 6 mm thresholds of attachment loss (AL) ranged from 0.52 to 0.66 during 2011–2014. When evaluating inter-examiner agreement using information collected from three periodontal sites for comparability to the NHANES 2003–04 periodontal examination protocols, Kappa scores for combined moderate-severe periodontitis was 0.65 and 0.80 with percent agreement 84 and 92% during 2011–2014.

The assessment of inter-examiner reliability for key continuous measures of periodontal status, such as mean AL, mean PD, and mean recession (CJ) are presented in Table 5. For overall attachment loss and pocket depth across all periodontal sites, the inter-class coefficients (ICCs) ranged from 0.80–0.90 and 0.79–0.86 respectively during 2011–2012 and 2013–2014. The ICCs ranged from 0.88 to 0.96 for gingival recession during the same period. Overall, for attachment loss the ICCs for facial measures ranged from 0.78 to 0.86 and for the lingual measures ranged from 0.80 to 0.89 during 2011–2014. For pocket depth, the ICCs values were similar for attachment loss. However, the overall ICCs for the mid-site pocket depth measures were 0.55 and 0.70 for mid-facial and were 0.66 and 0.80 for mid-lingual. When using information collected from only the three facial periodontal sites for comparability to the NHANES 2003–04 protocols, the ICCs for mean AL was 0.83 and 0.85 and mean pocket depth was 0.82 and 0.86 during 2011–2014.

**Table 5:** Dental examiner inter-class correlation coefficients (ICCs) for selected periodontal measures, National Health and Nutrition Examination Survey (NHANES) 2011–2014

	Periodontal Measures	NHANES 2011–2012				NHANES 2013–2014				NHANES 2011–2014			
**#**		n	Ref Mean	Exam Mean	ICC	n	Ref Mean	Exam Mean	ICC	n	Ref Mean	Exam Mean	ICC
A	AL mean – 6 sites^1^	25	1.87	2.28	0.80	38	1.39	1.60	0.90	63	1.58	1.87	0.86
B	AL mean – 6 sites^1^	35	1.30	1.54	0.88	51	1.57	1.71	0.83	86	1.46	1.64	0.85
A	PD mean – 6 sites^1^	25	1.56	1.79	0.83	38	1.06	1.17	0.82	63	1.26	1.42	0.86
B	PD mean – 6 sites^1^	35	1.25	1.42	0.79	51	1.29	1.42	0.86	86	1.28	1.42	0.84
A	CJ mean – 6 sites	25	−0.31	−0.49	0.91	38	−0.33	−0.43	0.95	63	−0.32	−0.45	0.93
B	CJ mean – 6 sites	35	−0.05	−0.12	0.96	51	−0.28	−0.29	0.88	86	−0.19	−0.22	0.90
A	AL Mean - distal facial	25	1.92	2.21	0.79	38	1.45	1.58	0.86	63	1.64	1.83	0.84
B	AL Mean - distal facial	35	1.40	1.44	0.93	51	1.69	1.77	0.68	86	1.57	1.63	0.78
A	AL Mean - mid facial	25	1.48	1.87	0.74	38	1.21	1.40	0.93	63	1.31	1.58	0.86
B	AL Mean - mid facial	35	1.05	1.46	0.77	51	1.35	1.49	0.83	86	1.23	1.48	0.81
A	AL Mean – mesial facial	25	1.90	2.23	0.72	38	1.47	1.62	0.85	63	1.64	1.86	0.81
B	AL Mean – mesial facial	35	1.37	1.44	0.90	51	1.70	1.68	0.74	86	1.57	1.59	0.81
A	AL Mean - distal lingual	25	2.10	2.62	0.76	38	1.50	1.79	0.79	63	1.74	2.12	0.80
B	AL Mean - distal lingual	35	1.45	1.68	0.84	51	1.74	1.94	0.80	86	1.62	1.83	0.81
A	AL Mean – mid lingual	25	1.72	2.18	0.84	38	1.22	1.42	0.91	63	1.42	1.72	0.89
B	AL Mean – mid lingual	35	1.13	1.55	0.78	51	1.31	1.56	0.81	86	1.23	1.56	0.80
A	AL Mean – mesial lingual	25	2.10	2.60	0.80	38	1.49	1.80	0.79	63	1.73	2.12	0.82
B	AL Mean – mesial lingual	35	1.42	1.68	0.84	51	1.65	1.82	0.83	86	1.56	1.76	0.84
A	PD Mean - distal facial	25	1.81	1.83	0.88	38	1.27	1.26	0.84	63	1.49	1.48	0.86
B	PD Mean - distal facial	35	1.52	1.45	0.85	51	1.52	1.58	0.78	86	1.52	1.53	0.81
A	PD Mean - mid facial	25	0.78	1.21	0.65	38	0.38	0.66	0.63	63	0.54	0.88	0.70
B	PD Mean - mid facial	35	0.49	1.08	0.28	51	0.57	0.88	0.72	86	0.53	0.96	0.55
A	PD Mean – mesial facial	25	1.82	1.91	0.86	38	1.31	1.34	0.85	63	1.52	1.57	0.88
B	PD Mean – mesial facial	35	1.50	1.45	0.79	51	1.55	1.59	0.80	86	1.53	1.53	0.80
A	PD Mean - distal lingual	25	1.93	2.15	0.86	38	1.37	1.43	0.78	63	1.59	1.72	0.87
B	PD Mean - distal lingual	35	1.63	1.66	0.81	51	1.64	1.74	0.85	86	1.63	1.71	0.84
A	PD Mean – mid lingual	25	1.11	1.53	0.70	38	0.68	0.87	0.74	63	0.85	1.13	0.76
B	PD Mean – mid lingual	35	0.81	1.23	0.55	51	0.88	1.05	0.74	86	0.85	1.13	0.66
A	PD Mean – mesial lingual	25	1.90	2.11	0.80	38	1.37	1.49	0.75	63	1.58	1.73	0.82
B	PD Mean – mesial lingual	35	1.58	1.65	0.83	51	1.63	1.70	0.85	86	1.61	1.68	0.84
A	AL mean – 3 sites^2^	25	1.76	2.10	0.76	38	1.38	1.53	0.91	63	1.53	1.76	0.85
B	AL mean – 3 sites^2^	35	1.27	1.45	0.90	51	1.58	1.65	0.79	86	1.46	1.57	0.83
A	PD mean – 3 sites^2^	25	1.47	1.65	0.83	38	0.99	1.09	0.83	63	1.18	1.31	0.86
B	PD mean – 3 sites^2^	35	1.16	1.32	0.78	51	1.21	1.35	0.84	86	1.19	1.34	0.82

Notes

[1]: overall mean is for the combined mesio-, mid-, and disto-facial plus the mesio-, mid-, and disto-lingual periodontal sites

[2]: overall mean is for the combined mesio-, mid-, and disto-facial periodontal sites (NHANES 2003–04 examined sites)

[#]: Number of paired observations

[n]: Number of replicate exams

[#]: Dental examiner

[AL]: Attachment loss

[PD]: Pocket depth

[3 sites]: Based on partial-mouth design used in earlier NHANES

## Discussion

Oral health data from NHANES is used for two important purposes: as the principle source of information for surveillance of dental diseases and OH related issues at the national level and to explore potential associations between OH status and other conditions. During the 2011–2014 NHANES, the oral health component was modified to make the oral assessments comparable to many procedures utilized during 1999–2004. Unlike 2005–2010, where the OH examination was administered by a registered health technologist or dental hygienist, beginning in 2011 the OH examination was administered by a dentist. In 2011, the assessment for dental caries at the tooth-surface level and an evaluation for dental fluorosis were re-introduced and are comparable to the procedures used during 1999–2004. In 2013, a digital imaging process was added to explore the utility of assessing dental fluorosis indirectly—through images, rather than a direct clinical assessment [5, 16]. The full mouth periodontal examination (FMPE) was continued during 2011–2014 producing six years of continuous FMPE data.

This is the fifth paper in a series of methodology reports that cover 16 years of OH data collection since the introduction of the continuous NHANES in 1999 [1, 4, 8, 15]. The information is organized and presented to facilitate comparisons between the various survey periods, for example, when comparing examiner reliability for determining dental caries experience from 2001 to 2004 to 2011–2014, or using examiner data from the FMPE to calculate partial mouth periodontal examiner reliability statistics for 2011–2014 to permit comparison to previously reported statistics from 2003 to 2004. As with prior papers, two important measures of examiner reliability, inter-class correlation coefficients for continuous data and Kappa statistics for categorical data, were examined to compare data reliability across multiple years. Although percent (proportion) agreements are presented, Kappa statistics are typically preferable to better differentiate between examiners as a measure for reliability. There is a subtle difference between the concepts of reliability and agreement. Reliability is a ratio of variability and agreement is a proportion of identical ratings [17]. An alternate concept behind the preference for Kappa over percent agreement is that the Kappa computation corrects the agreement for what would be expected by chance alone [18]. This mathematical adjustment usually produces a value that is lower compared to the basic calculation for percent agreement, but when the expected agreement is due to greater chance (guessing), the resulting lower Kappa calculation is also greater.

Guidelines proposed by Landis and Koch for interpreting kappa scores have been used by many researchers across many different health fields. To summarize Landis and Koch: a kappa statistic ≤0 is reflective of having “poor agreement”, a score of > 0 but ≤0.20 is “slight agreement”, 0.21–0.40 is “fair agreement”, 0.41–0.60 is “moderate agreement”, 0.61–0.80 is “substantial agreement”, and > 0.80 is “almost perfect agreement” [19]. To maintain consistency in interpretation with previous NHANES oral health data quality reports, we have used the guidelines suggested by Landis and Koch to evaluate examiner performance. An alternate, more “user-friendly” approach to interpreting Kappa is the use of 0.60 as a threshold, where any Kappa below 0.60 suggests inadequate reliability between an examiner and the reference [20]. Although the OH assessments implemented on the national health surveys are designed to minimize examiner subjectivity, some are inherently more difficult and more subjective than others.

Because the trainer and reference examiner was the same person, individual examiner agreement with the reference examiner was expected to be high. Examiner agreement was almost perfect for edentulism, tooth retention, retained root tips, and assessing third molars during 2011–2014. Examiner agreement was almost perfect for caries experience and untreated caries in primary and permanent dentition in 2011–2014. The one exception noted was for untreated caries in primary teeth, where substantial agreement was observed for Examiner A. Although examiner agreement was almost perfect for dental sealants during 2011–2014, there were differences between 2011 and 2012 and 2013–2014. Substantial agreement was observed for both examiners during 2011–2012, but improved to almost perfect agreement during 2013–2014.

Overall, when using data from the FMPE, there was substantial agreement among the examiners compared to the reference when CDC/AAP case definitions were used to assess for periodontal disease during 2011–2014. There was substantial agreement with the reference examiner by Examiner A during 2011–2012 and 2013–2014 for the key indicator of moderate-severe periodontitis. However, Examiner B represents a different examiner for 2011–2012 and 2013–2014 and there were significant differences in examiner performance between both examiners for this key indicator. The Kappa scores for moderate-severe periodontitis were 0.55 and 0.79, which suggests better agreement existed between the reference examiner and the 2013–2014 Examiner B compared to the 2011–2012 Examiner B. Using a Kappa threshold of 0.60 to assess examiner reliability, there was adequate reliability among examiners with the reference examiner across most periodontal indicators evaluated during 2011–2014.

To assess examiner reliability when using continuous measures of periodontal status, ICCs were calculated. The basic formula construct for the computation of an ICC is: reliability = variability between examiners / (variability between examiners + measurement error). The resulting statistic will range from 0 to 1, the range implying completely unreliable to perfect reliability [21]. An important consideration when interpreting ICCs is examiner bias or measurement error. As measurement error approaches the same value as examiner variability, the calculated ICC becomes closer to 0.5. When utilizing ICCs in dental research, it has been suggested that a threshold of 0.75 or greater would represent excellent reliability [22]. For the 2011–2014 periodontal data, the mean attachment loss and pocket depth ICC statistics as measured for all six sites around all teeth was 0.84 and 0.86 for each examiner, indicating excellent overall reliability for AL and PD. The ICC statistics for recession were also excellent (0.90 and 0.93). When evaluating the individual sites, the mid-facial and mid-lingual pocket depth sites were the most challenging for reproducibility among the examiners, specifically Examiner B (2011–2012) with the reference. For Examiner B, the ICC statistics were 0.28 (mid-facial) and 0.55 (mid-lingual). Although the ICCs from these two sites indicate that data quality is less than optimal, information obtained from the mid-tooth sites are not used to derive the CDC/AAP case definitions for periodontitis.

The difference between examiners with regards to performance is most striking for the mid-tooth sites. Across both data collection periods, the ICC statistics for Examiner A for the mid-facial PD was 0.65 and 0.66; 0.70 and 0.74 for mid-lingual PD. Examiner B during 2013–2014 was more comparable with Examiner A. Interestingly, during 2009–2010, the tooth sites presenting the greatest challenge to data reliability for pocket depth measures were again the mid-facial and the mid-lingual periodontal sites. During this period, the examiners were not dentists, but dental hygienists, and the ICC statistics ranged from 0.15 to 0.59 for both periodontal sites. In contrast, for both examiners during 2009–2010, the calculated ICC statistics for overall mean attachment loss for all six sites around all teeth was 0.80 and 0.88, indicating excellent overall reliability for AL. The performance of examiners between 2009 and 2010 (dental hygienists) and 2011–2014 (dentists) were remarkably similar for overall mean AL with ICCs ranging from 0.80 to 0.90. There was considerable consistency when comparing each examiner (both dental hygienists and dentists) with the reference for detecting moderate and severe periodontitis from 2009 to 2014 as well. For example, there was adequate reliability during 2009–2010 (0.70 and 0.71) and during 2011–2014 (0.66 and 0.69) among examiners when detecting moderate and severe periodontists using a Kappa threshold of 0.60 as a benchmark.

When summarizing assessments involving periodontal status, the Kappa statistics for the three main examiners during 2011–2012 and in 2013–2014 ranged from .45 to 1.0 and inter-class coefficients ranged from 0.28 to 0.96. Generally, achieving greater inter-examiner reliability was better for clinical recession compared to pocket depth and the area of greatest challenge was measuring pocket depth at the mid-facial and mid-lingual sites. Because pocket depth is an essential component for calculating attachment loss, aggregate measures of periodontitis can be affected by increase variability in inter-examiner reliability when examiners are not consistent across all measured sites. Our findings suggests that if these sites are to be measured in future epidemiologic studies for periodontal status, additional measures for training and monitoring should be considered to reduce potential measurement error and to facilitate examiner reliability.

When comparing 2013–2014 examiner reliability to 2003–2004, examiner performance as measured by Kappa appears to be generally similar. Examiners from 2013 to 2014 appear to have slightly higher agreement with the reference when detecting untreated caries in primary and permanent teeth; and similar agreement when assessing for tooth retention and dental sealants. When using comparable methodology assessing periodontal status at 3 dental sites characterized by the standard partial-mouth periodontal examination administered during 2003–2004, examiners from 2003 to 2004 and 2013–2014 performed similarly. Overall, examiners from both periods assessed periodontal disease with adequate reliability (Kappa > 0.60) and reliability was excellent for mean loss of attachment (ICC > 0.75). However, reliability was less than optimal for mean probing depth for Examiner A during 2003–2004 (ICC < 0.75).

There are several important considerations for analysts using the NHANES oral health data. Regarding measurement of oral diseases and conditions, data collection methodology has periodically changed between 1999 and 2014. During 1999–2004, dental caries followed a comprehensive, dental surface-level assessment for each tooth and periodontal assessments were made using a partial-mouth examination. From 2005 to 2008, a Basic Screening Examination (BSE) assessing dental caries and sealants was utilized and this was administered by health technologists. During 2009–2010, dental hygienists conducted a full-mouth periodontal examination (FMPE) and the BSE. Between 2011 and 2014, the dental examiners were dentists and they conducted a FMPE and a comprehensive assessment for dental caries using protocol comparable to 1999–2004. Evaluating examiner reliability represents one element of quality assurance. Another element is assessing the validity of the method used to collect data. Although data collection methods periodically changed over the years, the expectation for accuracy remained high because accepted protocols were utilized. Analysts also should be cautious regarding interpretation of two-year OH estimates calculated from the continuous NHANES. In addition to sampling variations because of the survey design or methodology changes affecting data collection, insufficient sample size for some subgroups could produce statistically unreliable estimates. Researchers should routinely evaluate whether the denominator count is > 30 and the relative standard error is less than 30%. For most OH calculations, using four years of data for analyses will reduce the effect of sampling variation between the two two-year periods and produce more accurate estimates.

## Conclusion

Overall, findings from this report indicate that the assessments of OH data reliability, for NHANES 2011 to 2014, are considered to reflect at least substantial agreement for tooth count and dentition. For periodontal disease the majority of assessments showed at least moderate agreement but some assessments fell below what would be considered adequate agreement (< 0.6 kappa value). Evaluations of inter-examiner reliability for the FMPE conducted between 2009 to 2014 indicate adequate reliability among the examiners (both dental hygienists and dentists) when compared to the reference for several measurement indicators of periodontal status, including the detection of moderate and severe periodontitis as well as mean AL and PD. The main area of concern is measurements made of a tooth at the mid-facial and to a lesser extent, measurements made at the mid-lingual site. When evaluating examiners during 2011 to 2014 and from 2003 to 2004, comparable reliability among examiners exists. Finally, the 2011–14 QA findings presented in this report add to the continuous quality monitoring of the OH examination on NHANES. These inter-examiner findings provide insight into data reliability monitoring in a large-scale ongoing epidemiologic study.

## Abbreviations

CDC/AAP: Centers for Disease Control and American Academy Periodontology
DMFT: Number of decayed, missing, and filled teeth
ICC: intra-class correlation coefficient
NHANES: National Health and Nutrition Examination Survey
OH: Oral health
QA: Quality Assurance
